# Trends in Tuberculosis Incidence and Mortality in South Africa and Bulgaria (2000–2023): The Impact of Income, Poverty, Unemployment, and Universal Health Coverage

**DOI:** 10.3390/epidemiologia7020039

**Published:** 2026-03-04

**Authors:** Siyabonga Kave, Joana Simeonova, Antoniya Yanakieva, Alexandrina Vodenitcharova, Denisha Govender, Yandisa Sikweyiya, Nelisiwe Khuzwayo

**Affiliations:** 1 School of Nursing and Public Health, College of Health Sciences, Howard College Campus, University of KwaZulu-Natal, Durban 4041, South Africa; denishagovender4@gmail.com (D.G.); yandisa.sikweyiya@mrc.ac.za (Y.S.); khuzwayone@ukzn.ac.za (N.K.); 2 Department of Social and Preventive Medicine and Disaster Medicine, Faculty of Public Health, Medical University, 1527 Sofia, Bulgaria; 3 Department of Health Technology Assessment, Faculty of Public Health, Medical University, 1527 Sofia, Bulgaria; a.yanakieva@foz.mu-sofia.bg; 4 Research Institute of Innovative Medical Science, Medical University, 1527 Sofia, Bulgaria; 5 Department of Bioethics, Faculty of Public Health, Medical University, 1527 Sofia, Bulgaria; a.vodenicharova@foz.mu-sofia.bg; 6 Gender and Health Research Unit, South African Medical Research Council, 1 Soutpansberg Road, Pretoria 0084, South Africa

**Keywords:** tuberculosis, TB incidence, TB mortality, South Africa, Bulgaria, social determinants of health, unemployment, health system coverage

## Abstract

Background: Tuberculosis (TB) remains a major global public health challenge, with substantial variation across countries. South Africa has one of the highest TB incidence and mortality rates globally, while Bulgaria, a low-incidence country, faces a persistent TB burden among vulnerable populations. Objectives: To compare national trends in TB incidence and mortality in South Africa and Bulgaria from 2000 to 2023 and explore associations with selected socioeconomic indicators and health system coverage. Methods: An ecological, descriptive, analytical study used national-level data from the WHO, World Bank, and official statistics. TB trends were analyzed alongside income, poverty, unemployment, and Universal Health Coverage indicators. Time series measures and Pearson correlation were used descriptively to summarize co-variation over time. Results: Between 2000 and 2023, TB incidence declined by approximately 44% in the Republic of South Africa and 69% in Bulgaria. In both countries, TB incidence co-varied strongly with unemployment (RSA: r = 0.805; BG: r = 0.723). In Bulgaria, TB incidence was also strongly negatively associated with GDP per capita (r = −0.910), whereas no significant association with GDP was observed in South Africa. These findings indicate that TB trends co-varied more closely with labour market conditions in both contexts, while broader economic growth co-occurred with declining TB incidence only in Bulgaria. Conclusions: TB trends co-occurred with changes in socioeconomic conditions and health system coverage, with differing patterns across contexts. Findings highlight the relevance of equity-oriented, context-specific TB control strategies integrated with social and economic policies.

## 1. Background

Tuberculosis (TB) remains a major global infectious disease with profound social, economic, and health implications, persisting throughout human history [1]. Evidence indicates that TB has affected human populations for more than 70,000 years and continues to be associated with substantial global burden, with an estimated two billion people infected worldwide [2]. Importantly, TB does not affect populations uniformly; higher TB burden is observed among individuals and communities experiencing socioeconomic disadvantage, marginalization, and limited access to health services [3].

In 2023, an estimated 8.2 million people were newly diagnosed with TB globally [1,2], predominantly among adults, with men accounting for a higher share of cases and deaths than women [4]. Sub-Saharan Africa (SSA) remains the region with the highest TB burden, reporting an incidence of approximately 201 cases per 100,000 population [1,5]. Despite the availability of effective treatment, TB was associated with an estimated 1.25 million deaths in 2023, with mortality concentrated among people living with Human Immunodeficiency Virus (HIV) and those experiencing delayed diagnosis or treatment interruption [6]. Untreated TB has been reported to have a case fatality rate of up to 50%, highlighting the clinical importance of timely detection and sustained access to care [2,7].

Adherence to the World Health Organization (WHO) recommended 4–6-month treatment regimen has been shown to achieve cure rates of approximately 85% under programmatic conditions [1]. However, biomedical effectiveness alone may be insufficient to fully account for TB patterns at the population level [8]. Approximately half of individuals affected by TB incur catastrophic costs related to diagnosis and treatment, including direct medical expenses and indirect costs such as income loss and transport [1,2]. In this context, progress towards TB control is closely associated with broader health system performance, universal health coverage (UHC), and the availability of social protection mechanisms [9,10]. Addressing social determinants of health (SDoH), including poverty, undernutrition, unemployment, substance use, HIV, diabetes, stigma, and health literacy, is therefore widely discussed in the literature as relevant to sustainable TB control efforts [11].

Tuberculosis both reflects and is embedded within broader patterns of social inequality. Overcrowded living conditions, food insecurity, unemployment, and limited access to healthcare have been consistently described in the literature alongside higher TB burden and adverse treatment-related outcomes [3,12]. Gender disparities further shape TB epidemiology, as men globally experience higher TB incidence and mortality, with a male-to-female incidence ratio of approximately 1.8 [13,14]. These patterns underscore the relevance of situating TB trends within their broader socioeconomic and health system contexts.

Against this backdrop, the Republic of South Africa (RSA) and Bulgaria (BG) represent contrasting yet analytically informative TB settings. These countries were selected as analytically divergent case studies to explore how national-level TB trends co-vary with selected socioeconomic indicators across markedly different epidemiological, health system, and socioeconomic contexts. The RSA represents a high TB and HIV burden setting within an upper-middle-income economy, while BG represents a low-incidence European Union member state shaped by different historical, demographic, and health system trajectories. This contrast is not intended to imply comparability in causal pathways or policy transferability, but rather to examine whether broad socioeconomic alignments with TB trends are observable across heterogeneous contexts. Using divergent cases allows exploration of whether similar social patterning of TB incidence is evident across settings with distinct epidemic profiles and structural conditions.

RSA is among the 30 highest TB-burden countries globally, accounting for approximately 3% of global TB cases in 2023, with a high prevalence of HIV co-infection (approximately 55%) [15,16]. Although classified as an upper-middle-income country [17], RSA continues to experience extreme income inequality, high unemployment, and persistent gaps in equitable health service delivery, which characterize the broader social and structural context within which TB transmission and outcomes are observed [18,19].

In contrast, BG is a low-incidence, high-income European Union member state, contributing a relatively small share of global TB cases. HIV prevalence among TB patients in Bulgaria is substantially lower than in RSA, and HIV/TB co-infection plays a less prominent role in national TB epidemiology [8,19]. Nevertheless, TB remains concentrated among specific vulnerable populations, including Roma communities, older adults, and socially excluded groups, occurring alongside enduring socioeconomic disparities, population ageing, and barriers to healthcare access [19,20].

Comparing these two countries allows examination of whether similar socioeconomic and health system indicators, such as income, poverty, unemployment, and UHC service coverage, are associated with TB trends under markedly different epidemiological, demographic, and structural conditions. RSA represents a high-burden, HIV-endemic setting characterized by deep structural inequality, while BG represents a low-incidence, ageing European context with lower HIV prevalence but persistent social exclusion. This contrast provides an analytical basis for assessing how TB trends align with measures of social vulnerability across divergent health systems and socioeconomic environments.

Accordingly, this study analyses national-level trends in TB incidence and mortality in RSA and BG from 2000 to 2023 and explores their associations with selected socioeconomic indicators and UHC service coverage. By adopting a comparative, ecological perspective, the study aims to contribute to understanding how social determinants and health system factors co-vary with TB trends across diverse settings, informing context-sensitive policy and programme responses.

## 2. Methods

### 2.1. Study Design

This study employed an ecological, descriptive–analytical, longitudinal design based on secondary national-level data to examine trends in TB incidence and mortality in RSA and BG between 2000 and 2023, and their associations with selected socioeconomic and health system indicators. Given the ecological nature of the data, the analysis was exploratory and descriptive, and no causal inferences were intended or drawn. This design was chosen to describe long-term national patterns and co-occurring trends rather than to test hypotheses or establish causal relationships, given the ecological and time-series nature of the data.

### 2.2. Data Sources

The TB incidence and mortality data were obtained from the WHO Global Tuberculosis Database (2000–2023), which adjusts for under-detection and misclassification. Socioeconomic indicators were retrieved from the World Bank World Development Indicators database [21], while national poverty and unemployment statistics were sourced from Statistics South Africa and the Bulgarian National Statistical Institute. The indicators analyzed included:

TB incidence per 100,000 populations (estimated incidence);

TB mortality per 100,000 populations (estimated mortality);

Gross Domestic Product (GDP) per capita (current USD);

Poverty measures (national poverty lines);

Unemployment rate (% of labour force);

Universal Health Coverage Service Coverage Index (UHC SCI).

All data were aggregated at the national level and analyzed annually.

Comparable longitudinal data on TB–HIV co-infection were not available for BG across the full study period; HIV is therefore discussed contextually for the RSA only and was not included as a comparative analytical variable.

### 2.3. Socioeconomic Indicators

Due to incomplete and inconsistent availability of data based on the international poverty line across the full study period, national poverty thresholds were used for each country. National poverty lines are defined and regularly updated by official statistical authorities and are considered more appropriate for within-country temporal analysis.

During the most recent reporting period, the average monthly poverty line was BGN 637.92 (USD 370.22) per person in BG and ZAR 760 (USD 42.24) per person in RSA. The unemployment rate was defined as the proportion of the labour force without work but actively seeking employment, consistent with International Labour Organization (ILO) standards.

The Universal Health Coverage Service Coverage Index (UHC SCI), developed by the WHO and World Bank, was used as a composite indicator of essential health service coverage. The index combines 14 tracer indicators across four domains and ranges from 0 to 100, with higher values indicating broader service coverage [9].

### 2.4. Trend and Time-Series Analysis

Time-series analysis was conducted to describe long-term trends in TB incidence and mortality. Absolute change was calculated as the difference between the rate in year i (y_i_) and either a fixed reference year (y_0_, year 2000) or the immediately preceding year (y_i−1_), using the following expressions:

Fixed-base absolute change:

Δ_i_/_0_ = y_i_ − y_0_

Chain-base absolute change:

Δ_i_/_i−1_ = y_i_ − y_i−1_

Dynamic indices were calculated to express relative change over time:

Fixed-base index:

I_i_/_0_ = (y_i_/y_0_) × 100

Chain-base index:

I_i_/_i__−1_ = (y_i_/y_i−1_) × 100

Growth rates were derived as:

Fixed-base growth rate:

R_i_/_0_ = [(y_i_ − y_0_)/y_0_] × 100

Chain-base growth rate:

R_i_/_i−1_ = [(y_i_ − y_i−1_)/y_i−1_] × 100

where y_i_ represents the TB rate in year i, y_0_ the rate in the baseline year (2000), and y_i−1_ the rate in the previous year. Fixed-base changes compare each year to 2000, while chain-base changes compare consecutive years. These measures were used to describe temporal patterns rather than to predict future outcomes. No formal time-series modeling (e.g., autoregressive or trend-adjusted regression) was conducted, as the primary aim was descriptive comparison rather than statistical inference

### 2.5. Correlation Analysis

Prior to correlation analysis, variables were assessed for distributional properties using the Shapiro–Wilk test. Deviations from normality were observed for TB incidence, TB mortality, national poverty measures, and unemployment rates in at least one of the two countries (Shapiro–Wilk *p*-values ranged from <0.01 to 0.04 across indicators and years), while GDP per capita and UHC SCI approximated normal distributions more closely in later years of the series. In addition, all variables exhibited pronounced long-term temporal trends.

Pearson correlation coefficients were used descriptively to summarize linear co-variation over time, rather than to test statistical dependence or infer causal relationships. Correlations were interpreted as indicators of parallel or divergent temporal trajectories between variables, not as evidence of causal or population-level associations.

Given the potential for temporal autocorrelation and shared underlying trends in long time-series data, correlation coefficients and associated *p*-values were interpreted cautiously and used only to summarize the direction and relative strength of co-occurring trends. To assess the robustness of findings to distributional assumptions, Spearman rank correlation coefficients were calculated as a sensitivity analysis; the direction and relative magnitude of associations were consistent across Pearson and Spearman methods. Pearson correlations are presented for ease of interpretation and comparability with prior ecological TB studies.

No adjustments were made for serial correlation or time-dependent confounding, and findings are presented descriptively to illustrate broad temporal co-variation between TB indicators and selected socioeconomic measures.

### 2.6. Missing Data and Reproducibility

Missing data were not imputed due to the risk of introducing bias in long-term national time-series, particularly where missingness was non-random or limited to specific socioeconomic indicators. TB incidence and mortality data were obtained as complete national estimates from the WHO Global Tuberculosis Database, which applies standardized adjustment methods to account for under-detection and reporting uncertainty.

Analyses were therefore conducted using available observations for socioeconomic variables, and patterns of missingness were assessed descriptively and reported where relevant. All data originated from publicly accessible international and national databases, and a detailed list of data sources and indicator definitions is provided to support transparency and reproducibility. All statistical analyses were performed using SPSS version 25.0.

## 3. Results

### 3.1. Country Context

#### 3.1.1. RSA

The RSA has the largest economy in Africa and is classified as an upper-middle-income country, with a gross domestic product (GDP) per capita of USD 6002.5 in 2023 [17]. Despite this economic status, the period was characterized by structural challenges, including electricity shortages, limited job creation, and transport and logistics bottlenecks [22]. These conditions co-occurred with persistently high income inequality and unemployment, variables that have been described in the literature in relation to population health patterns, including TB and health service utilization [5].

#### 3.1.2. BG

Over the past three decades, BG has undergone substantial political and economic transition and has been a member of the European Union since 2007 [9]. In 2023, BG transitioned from upper-middle-income to high-income status according to World Bank classifications [7]. Despite this progress, marked social and regional inequalities persisted over the study period, including disparities in employment, income, and access to health services, particularly among marginalized populations.

Comparing RSA and BG highlights differences in epidemiological and social contexts alongside observed TB trends. In RSA, the high burden of HIV co-infection (~55% among TB patients) and persistent challenges with MDR-TB co-occurred with the pronounced rise in TB incidence during the early 2000s and the slower decline in mortality compared with BG. These biomedical challenges co-occurred with entrenched social vulnerabilities, including high poverty levels, unemployment, and income inequality, which have been described alongside patterns of health service access and treatment continuity in prior studies [23]. In contrast, BGs exhibited lower HIV prevalence and a more limited MDR-TB burden alongside more consistent reductions in TB incidence and mortality. However, persistent unemployment and marginalization of vulnerable groups, such as Roma communities and socially excluded populations, continued to be observed alongside residual TB burden. Together, these patterns indicate that TB trends in both countries occurred alongside differing biomedical and socioeconomic contexts, underscoring the value of a comparative perspective that integrates epidemiological and contextual indicators rather than presenting country-specific information in isolation.

### 3.2. Trends in TB Incidence and Mortality (2000–2023)

Table 1 presents national estimates of TB incidence and mortality in RSA and BG between 2000 and 2023. Substantial differences in TB burden and long-term trends are evident between the two countries.

Table 1 presents contrasting long-term TB trends, with BG displaying a steady decline in both incidence and mortality, while RSA displayed a sharp rise in the early 2000s followed by a sustained decline after 2008.

The COVID-19 pandemic coincided with substantial disruptions to health services globally, including TB case detection and treatment continuity. Although WHO mortality and incidence estimates incorporate adjustments to account for under-detection and reporting disruptions during this period, short-term fluctuations observed after 2020 should be interpreted cautiously. Accordingly, greater emphasis is placed on long-term trends rather than year-to-year changes during the pandemic.

### 3.3. Time-Series Description of TB Incidence

#### 3.3.1. Absolute Change

Time-series indicators show that BG’s TB incidence declined consistently across most years after 2003, reflecting sustained reductions over time. In RSA, declines emerged later and were more uneven, with the most pronounced reductions occurring after 2015.

#### 3.3.2. Dynamic Index and Growth Rate

Dynamic index analysis describes contrasting trajectories between the two countries (Table 2).

In RSA, TB incidence increased to 166.7% of the 2000 baseline by 2008, followed by a sustained decline to 56% of baseline levels by 2023. In BG, TB incidence declined more consistently, reaching approximately 31% of baseline levels by 2023.

Overall, TB incidence declined by 43.9% in RSA and 69.2% in BG between 2000 and 2023, reflecting a larger proportional decline over the study period in BG compared to RSA.

### 3.4. Social Determinants of TB

**Figure 1:**
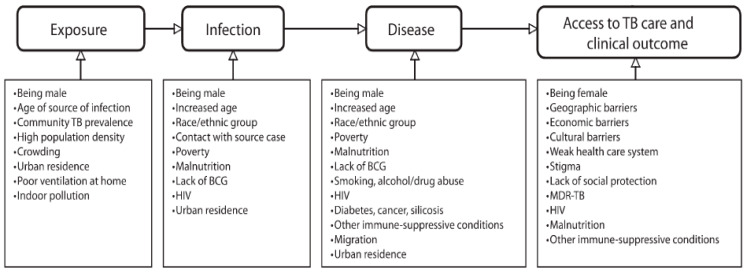
Conceptual framework summarizing selected social and economic factors discussed in the literature in relation to different stages of TB vulnerability and care. The figure is illustrative and does not represent empirical findings from the present analysis. Abbreviations: TB, tuberculosis; BCG, Bacillus Calmette–Guérin; HIV, human immunodeficiency virus; MDR-TB, multidrug-resistant tuberculosis.

Figure 1 summarizes key social determinants discussed in the literature review in relation to different stages of TB vulnerability, including exposure, disease progression, access to care, and treatment continuity. These factors provide a conceptual framework for interpreting the observed TB trends, particularly patterns of co-variations with unemployment and poverty identified in the correlation analysis.

Risk factors for different stages of TB pathogenesis and epidemiology [24,25].

Poverty, undernutrition, food insecurity, overcrowding, and poor ventilation have been described in the literature alongside higher TB burden, while stigma, income loss, and transport barriers have been discussed in relation to delays in health-seeking and continuity of care [15,26].

Table 3 summarizes selected socioeconomic indicators relevant to TB epidemiology in RSA and BG from 2000 to 2023, including GDP per capita, poverty measures, unemployment rates, and UHC service coverage.

### 3.5. Associations Between TB Incidence and Selected Social Determinants

Pearson correlation analysis was used to explore associations between TB incidence trends and selected socioeconomic indicators at the national level (Table 4).

As presented in Table 4, TB incidence demonstrated strong positive co-variation with unemployment in both countries. In RSA, TB incidence also co-varied with national poverty levels, while GDP per capita showed no significant relationship. In contrast, BG displayed a strong negative association between TB incidence and GDP per capita, alongside a positive association with unemployment. These patterns indicate closer co-variation between TB incidence trends and labor market indicators in both contexts, while broader economic indicators displayed differing relationships across countries.

Given the ecological study design and the presence of long-term temporal trends, these correlations are interpreted as associations between co-occurring trends rather than evidence of causal effects.

Overall, TB incidence trends in both countries co-varied with selected socioeconomic indicators, although the direction and strength of associations differed across contexts. These correlations describe parallel national-level trends over time and do not indicate causal relationships.

Pearson correlation analysis was applied descriptively to examine co-variation between TB mortality trends and selected socioeconomic indicators at the national level (Table 5). In RSA, TB mortality showed strong positive associations with unemployment and the proportion of the population living below the national poverty line, while the association with GDP per capita was weak and not statistically significant.

In BG, TB mortality declined steadily over the study period and demonstrated a strong negative association with GDP per capita, alongside a positive association with unemployment. As with incidence, these correlations reflect co-occurring long-term trends rather than causal relationships and should be interpreted cautiously given the ecological design and strong temporal patterning of the data.

These associations describe co-occurring national-level trends over time and do not imply causal or directional relationships.

## 4. Discussion

This comparative analysis examined long-term trends in TB incidence and mortality in RSA and BG between 2000 and 2023 and explored their associations with selected social determinants of health. While both countries experienced overall declines in TB burden during the study period, the magnitude, timing, and socioeconomic context of these trends differed substantially.

In BG, TB incidence and mortality declined steadily from the early 2000s onward. These trends were observed over a period that coincided with long-standing national TB control efforts, relatively high treatment success rates, and progressive integration of TB services into broader public health and social protection systems [24,27]. Over the same period, BG experienced economic growth, institutional consolidation following European Union accession, and gradual expansion of universal health coverage [11,28]. Within this broader context, the strong negative association observed between GDP per capita and TB incidence is consistent with the co-occurrence of improving aggregate economic indicators and declining TB rates. However, this relationship should be interpreted cautiously, as both variables exhibit strong long-term temporal trends and may capture broader structural change rather than a direct linkage.

Despite national-level progress, TB incidence in BG remained positively associated with unemployment. This pattern highlights persistent socioeconomic vulnerability within specific population groups, including Roma communities and people experiencing homelessness, where challenges related to stable employment, housing, and healthcare access have been documented [25]. These findings suggest that national averages may obscure localized or subgroup-specific patterns of TB burden.

In contrast, RSA experienced a persistently high TB burden throughout much of the study period. TB incidence and mortality increased sharply between 2000 and 2008, followed by a sustained decline from 2009 onward. These temporal trends were observed during a period characterized by expanded TB/HIV service integration, improvements in diagnostic capacity, and intensified case-finding activities [4,29]. Reductions in TB mortality occurred more gradually than declines in incidence and were observed alongside ongoing challenges related to multidrug-resistant TB, treatment interruption, and continuity of care [17].

Correlation analysis indicated strong positive associations between TB incidence and both unemployment and the proportion of the population living below the national poverty line in RSA. These findings describe TB trends that co-varied closely with indicators of socioeconomic disadvantage. By contrast, GDP per capita demonstrated a weak and non-significant association with TB incidence, suggesting that changes in aggregate national income did not parallel proportional changes in TB burden. This pattern aligns with documented levels of income inequality and uneven distribution of economic gains within the country [30,31].

Taken together, the comparative perspective indicates that TB trends occurred within distinct social and economic contexts in the two countries [32]. While BG’s declining TB burden was observed during a period of economic growth and relatively stronger social protection systems, RSA’s higher TB incidence co-occurred with persistent poverty, unemployment, and health system inequities. Importantly, these findings do not imply causality but rather describe how TB epidemiology aligns with broader structural conditions across different national settings [33].

### Limitations

This study has several important limitations. First, the analysis relied on national-level aggregate data, which may obscure substantial subnational and population-level heterogeneity in TB burden and social determinants. Regional disparities, urban–rural differences, and inequities affecting specific population groups could not be examined.

Second, the ecological design precludes causal inference. Associations identified through correlation analysis reflect co-occurring trends and may be influenced by unmeasured confounding factors or shared temporal patterns. The potential for ecological fallacy must therefore be acknowledged.

Third, data availability and completeness varied across indicators and years. Missing values for certain socioeconomic indicators may affect comparability and trend interpretation. TB incidence and mortality estimates produced by the World Health Organization were available throughout the COVID-19 pandemic period and incorporate adjustments for under-detection and reporting disruptions; nevertheless, short-term post-2020 fluctuations should be interpreted cautiously. In addition, reliance on secondary data introduces potential reporting bias, residual under-detection, and uncertainty in mortality attribution.

Finally, the use of correlation-based methods does not account for time-dependent confounding or structural breaks, and findings should be interpreted as descriptive rather than predictive or explanatory of underlying mechanisms.

## 5. Policy Implications and Recommendations

Although causal relationships cannot be inferred from this ecological analysis, the observed associations provide contextual insights that may inform TB policy discussions and programme planning.

The consistent positive association between TB incidence and unemployment in both countries highlights the relevance of labour-market conditions and social protection environments within which TB programmes operate. In BG, sustained declines in TB incidence alongside persistent associations with unemployment underscore the continued relevance of maintaining targeted, equity-oriented TB strategies. Continued outreach to socially excluded populations, including Roma communities, people without stable housing, and uninsured individuals, remains relevant within this context. Broader policies aimed at reducing regional socioeconomic disparities may align with efforts to engage with structural conditions that co-occur with higher TB incidence [8,34,35].

In RSA, strong associations between TB incidence, poverty, and unemployment are consistent with the relevance of situating TB responses within wider social and economic contexts. While biomedical interventions remain central, alignment between TB services and existing social protection, employment support, and welfare systems may be relevant for contextualizing barriers related to diagnosis, treatment initiation, and continuity of care [19,23]. Strengthening universal health coverage, particularly in under-resourced rural provinces, and improving continuity of care for people with drug-resistant TB remain important considerations within the national TB response [14].

Across both contexts, these findings support the relevance of multi-sectoral approaches that consider TB within broader social, labour, and welfare policy environments. Consideration of the socioeconomic context in which TB occurs may align with efforts to sustain gains achieved through improved diagnostics and treatment, while acknowledging that these relationships are complex, context-dependent, and not indicative of causal pathways [36].

## 6. Conclusions

This study examined national-level trends in TB incidence and mortality in RSA and BG between 2000 and 2023 and explored their co-variation with selected socioeconomic indicators. Both countries experienced overall declines in TB incidence during the study period, although the magnitude and timing of these trends differed substantially.

Between 2000 and 2023, TB incidence declined by approximately 44% in RSA and 69% in BG. In both countries, TB incidence co-varied strongly with unemployment over time. In RSA, TB incidence was also positively associated with the proportion of the population living below the national poverty line, while no significant association was observed with GDP per capita. In contrast, BG exhibited a strong negative association between TB incidence and GDP per capita, alongside a positive association with unemployment.

Taken together, these findings indicate that TB trends showed closer co-variation with labour market conditions in both settings, while broader economic growth co-occurred with declining TB incidence only in BG. Given the ecological design and use of national-level aggregate data, these associations reflect co-occurring population-level trends rather than causal relationships. Future research using subnational or individual-level data may help further elucidate how socioeconomic conditions intersect with TB epidemiology across diverse health system and policy contexts.

## Figures and Tables

**Table 1:** National-level estimated TB incidence and mortality rates per 100,000 population in the RSA and BG, 2000–2023.

RSA			BG		
Year	Incidence per 100,000	Mortality per 100,000	Year	Incidence per 100,000	Mortality per 100,000
2000	762	444	2000	52	4.0
2001	867	424	2001	61	4.0
2002	971	404	2002	53	4.0
2003	1070	385	2003	52	4.0
2004	1150	372	2004	49	4.0
2005	1210	357	2005	52	4.0
2006	1250	343	2006	51	4.0
2007	1270	330	2007	47	4.0
2008	1270	317	2008	49	3.0
2009	1260	304	2009	44	3.0
2010	1230	291	2010	41	3.0
2011	1200	279	2011	37	2.0
2012	1160	267	2012	36	2.0
2013	1110	255	2013	33	2.0
2014	1070	243	2014	32	2.0
2015	988	121	2015	28	2.0
2016	805	116	2016	27	2.0
2017	738	112	2017	25	1.0
2018	677	107	2018	23	1.0
2019	615	103	2019	23	1.0
2020	562	100	2020	16	1.0
2021	513	97	2021	12	1.0
2022	468	93	2022	14	1.4
2023	427	95	2023	16	1.4

**Table 2:** Descriptive time-series indicators of national TB incidence trends in the Republic of South Africa (RSA) and Bulgaria (BG), 2000–2023. Indicators summarize temporal change and do not imply causal relationships.

**RSA**							
**Year**	**Incidence (y_i_)**	**Δ_i_/_0_**	**Δ_i_/_i−1_**	**Index (2000 = 100)**	**Chain Index**	**Growth % (2000)**	**Growth % (y_i−1_)**
2000	762	–	–	100	100	–	–
2001	867	105	105	113.8	113.8	13.8	13.8
2002	971	209	104	127.4	111.9	27.4	11.9
2003	1007	245	36	132.2	103.7	32.2	3.7
2004	1150	388	143	150.9	114.2	50.9	14.2
2005	1210	448	60	158.8	105.2	58.8	5.2
2006	1250	488	40	164	103.3	64	3.3
2007	1270	508	20	166.7	101.6	66.7	1.6
2008	1270	508	0	166.7	100	66.7	0
2009	1260	498	−10	165.4	99.2	65.4	−0.8
2010	1230	468	−30	161.4	97.6	61.4	−2.4
2011	1200	438	−30	157.5	97.6	57.5	−2.4
2012	1160	398	−40	152.2	96.7	52.2	−3.3
2013	1110	348	−50	145.7	95.7	45.7	−4.3
2014	1070	308	−40	140.4	96.4	40.4	−3.6
2015	988	226	−82	129.7	92.3	29.7	−7.7
2016	805	43	−183	105.6	81.5	5.6	−18.5
2017	738	−24	−67	96.9	91.7	−3.1	−8.3
2018	677	−85	−61	88.8	91.7	−11.2	−8.3
2019	615	−147	−62	80.7	90.8	−19.3	−9.2
2020	562	−200	−53	73.8	91.4	−26.2	−8.7
2021	513	−249	−49	67.3	91.3	−32.7	−8.7
2022	468	−294	−45	61.4	91.2	−38.6	−8.8
2023	427	−335	−41	56	91.2	−43.9	−8.8
**BG**							
**Year**	**Incidence (y_i_)**	**Δ_i_/_0_**	**Δ_i_/_i−1_**	**Index (2000 = 100)**	**Chain Index**	**Growth % (2000)**	**Growth % (y_i−1_)**
2000	52	–	–	100	100	–	–
2001	61	9	9	117.3	117.3	17.3	17.3
2002	53	1	−8	101.9	86.9	1.9	−13.1
2003	52	0	−1	98.1	98.1	0	−1.9
2004	49	−3	−3	94.2	94.2	−5.8	−5.8
2005	52	0	−3	100	106.1	0	6.1
2006	51	−1	−1	98.1	98.1	−1.9	−1.9
2007	47	−5	−4	90.4	92.2	−9.6	−7.8
2008	49	−3	2	94.2	104.3	−5.8	4.3
2009	44	−8	−5	84.6	89.8	−15.4	−10.2
2010	41	−11	−3	78.8	93.2	−21.2	−6.8
2011	37	−15	−4	71.2	90.2	−28.8	−9.8
2012	36	−16	−1	69.2	97.3	−30.8	−2.7
2013	33	−19	−3	63.5	91.7	−36.5	−8.3
2014	32	−20	−1	61.5	96.9	−38.5	−3.0
2015	28	−24	−4	53.8	87.5	−46.2	−12.5
2016	27	−25	−1	51.9	96.4	−48.1	−3.6
2017	25	−27	−2	48.1	92.5	−51.9	−7.4
2018	23	−29	−2	44.2	92	−55.8	−8.0
2019	23	−29	0	44.2	100	−55.8	0
2020	16	−36	−7	30.8	69.6	−69.2	−30.4
2021	12	−40	−4	23.1	75	−76.9	−25.0
2022	14	−38	2	26.9	116.7	−73.1	16.7
2023	16	−36	2	30.8	114.3	−69.2	–

**Table 3:** Selected socioeconomic indicators relevant to TB epidemiology in RSA and BG. Correlation coefficients summarize co-variation between national-level time series and do not imply causality or directionality.

**RSA**					
**Year**	**Income (GDP per Capita in USD)**	**Poverty Ratio at $2.15 a Day (% Population)**	**% Population Below the National Poverty Line**	**Unemployment Rate (%)**	**UHC Service Coverage Index**
2000	3220	36.8	53	19.84	43
2001	2850	48.7	57	19.73	
2002	2690	57.3	48.5	19.66	
2003	4060	14	43.3	19.73	
2004	5220	n/a	55	19.63	
2005	8840	28.3	66.6	19.56	51
2006	6080	66.6	66.6	19.43	
2007	6590	n/a	47.6	19.39	
2008	6180	62.4	62.1	19.51	
2009	6370	n/a	39	20.51	
2010	7970	18	53.2	23.18	63
2011	8850	14	53.2	21.42	
2012	8080	n/a		21.79	
2013	7330	n/a		22.04	
2014	6860	20.5	55.5	22.61	
2015	6110	n/a	55.5	22.87	70
2016	5650	21.5	55.5	24.02	
2017	6620	21.5	55.5	23.99	71
2018	6910	47.6	55.5	24.22	
2019	6530	48.4	55.5	25.54	71
2020	5580	18.9	55.5	24.34	71
2021	6840	6.3	50	28.77	71
2022	6520	21.5	56.8	28.84	
2023	6020	21.5	55.5	27.99	79
**BG**					
**Year**	**Income (GDP per capita in USD)**	**Poverty ratio at $2.15 a day (% population)**	**% population below the national poverty line**	**Unemployment rate (%)**	**UHC service coverage index**
2000	1621	7.9		16.22	56
2001	1770	7.9	17.3	19.92	
2002	2090	7.9		18.11	
2003	2720	14	20.6	13.73	
2004	3390	10	21.7	12.04	
2005	3900	n/a	22	10.08	61
2006	4520	5.8	28.5	8.95	
2007	5890	1.8	16	6.88	
2008	7270	1.3	20.6	5.61	
2009	6990	1.3	20.7	6.82	
2010	6860	2	22.3	10.28	65
2011	7860	2.5	21.2	11.26	
2012	7430	2.3	21.2	12.27	
2013	7690	1.8	21	12.94	
2014	7910	1.7	21.8	11.42	
2015	7080	3.4	22	9.14	70
2016	7570	2	22.9	7.58	
2017	8380	1.4	23.4	6.16	72
2018	9440	0.9	22	5.21	
2019	9840	0.9	23.8	4.23	76
2020	10,200	0.2	22.1	5.13	73
2021	12,270	0.7	22.9	5.27	73
2022	13,640	9	20.6	4.27	
2023	15,890	0.7	30.3	4.3	

n/a—Not Available.

**Table 4:** Pearson correlation coefficients between TB incidence and selected socioeconomic indicators. Correlation coefficients summarize co-variation between national-level time series and do not imply causality or directionality.

	RSA	BG
Social Determinant	TB Incidence Trend	
GDP per capita	r = −0.246, *p* = 0.247, N = 24	r = −0.910 *, *p* = 0.001, N = 24
% Population below the national poverty line	r = 0.674 *, *p* = 0.001, N = 24	r = 0.378, *p* = 0.083, N = 24
Unemployment rate (%)	r = 0.805 *, *p* = 0.001, N = 24	r = 0.723 *, *p* = 0.001, N = 24

* Statistical significance correlation at *p* < 0.05. Correlation coefficients summarize co-variation between national-level time series and do not imply causality.

**Table 5:** Pearson correlation coefficients between TB mortality and selected socioeconomic indicators.

	RSA	BG
Social Determinant	TB Mortality Trend	TB Mortality Trend
GDP per capita	r = −0.312, *p* = 0.136, N = 24	r = −0.882 *, *p* = 0.001, N = 24
% Population below the national poverty line	r = 0.721 *, *p* = 0.001, N = 24	r = 0.402, *p* = 0.061, N = 24
Unemployment rate (%)	r = 0.846 *, *p* = 0.001, N = 24	r = 0.691 *, *p* = 0.001, N = 24

* Statistical significance correlation at *p* < 0.05.

## Data Availability

No new data were created or analyzed in this study. Data supporting the findings of this study are available in publicly accessible repositories and published sources, which are cited within the article.
